# Genome-Wide Identification and Characterization of the *LpSAPK* Family Genes in Perennial Ryegrass Highlight *LpSAPK9* as an Active Regulator of Drought Stress

**DOI:** 10.3389/fpls.2022.922564

**Published:** 2022-06-02

**Authors:** Jing Xing, Ruijie Zhao, Qing Zhang, Xinru Huang, Tingchao Yin, Jing Zhang, Bin Xu

**Affiliations:** College of Agro-Grassland Science, Nanjing Agricultural University, Nanjing, China

**Keywords:** perennial ryegrass, *LpSAPK* genes, abiotic stress, plant hormone, drought tolerance

## Abstract

*SAPK/SnRK2* family genes play crucial roles in plant growth, development, and abiotic stress responses. The objective of this study was to identify and characterize the *LpSAPK* genes in perennial ryegrass (*Lolium perenne* L.). The results showed that there are 10 *LpSAPKs* in perennial ryegrass that could be classified into three groups with similar genic (exon–intron) structures to their orthologous genes in Arabidopsis and other grass species. Ka/Ks analysis suggested that the *LpSAPKs* and their orthologs were under purifying selection to maintain their conserved function during evolution. Nine out of ten *LpSAPKs* were localized in the cytoplasm and nucleus with the exception of LpSAPK5 which was only observed in the cytoplasm. Most *LpSAPKs* were responsive to various abiotic stress and hormonal (ABA, cytokinin, and ethylene) treatments but were downregulated in leaves and upregulated in roots, suggesting that there were unknown *cis* elements in promoters of these genes or unidentified post-transcriptional mechanism responsible for the tissue-dependent stress-regulated expression of these *LpSAPKs*. Furthermore, *LpSAPK9* was identified as a candidate positive regulator in drought tolerance using a yeast ectopic expression system, and *LpSAPK9* showed contrasting expression changes in drought-sensitive and -tolerant ryegrass varieties, suggesting that expression levels of *LpSAPK9* were related to ryegrass drought tolerance. These results will facilitate further functional analysis of *LpSAPKs* for molecular breeding of ryegrass and other related grass species.

## Introduction

Protein phosphorylation and de-phosphorylation, catalyzed by kinases and phosphatases, comprise one important type of post-translational modification that plays in the regulation of plants’ adaptation to abiotic stresses (Couso et al., 2021). The stress-activated protein kinase (SAPK), also known as the SnRK2 (sucrose non-fermenting 1-related protein kinase 2 subfamily), is a plant-specific Ser/Thr protein kinase family that regulates plant growth, development, and abiotic stress responses *via* abscisic acid (ABA) dependent or independent signaling pathway (Pang et al., 2018; Chen et al., 2020b; Takahashi et al., 2020).

All SAPKs contain two domains: the N-terminal kinase domain and the C-terminal regulatory domain. The N-terminal kinase domain is conserved to the kinase domain of AMPK (AMP-activated protein kinase; Hrabak et al., 2003). The C-terminal domain consists of two subdomains, including Domain I and Domain II (Yoshida et al., 2006). The Domain I harbors about 30 amino acids, which exist in all SnRK2 members and are required for activation by osmotic stress; while the Domain II contains about 40 amino acids that mediate interaction with the clade A-type PP2Cs (2C Protein Phosphatases) for ABA signaling transduction (Kobayashi et al., 2004). Some SAPKs were identified as important regulators in plant tolerance to multi-environmental stresses. For example, in response to salt stress, AtSnRK2.4 and AtSnRK2.10 bind to phosphatidic acid (PA) through a 42 aa long domain, while the mutant proteins with the loss of PA interaction domains showed severe root inhibition under salt stress (Julkowska et al., 2015; Putta et al., 2020). Overexpression of *AtSnRK2.8* and *TaSnRK2.3/2.4/2.7* significantly enhanced drought stress tolerance in transgenic plants (Umezawa et al., 2004; Mao et al., 2010; Zhang et al., 2011; Tian et al., 2013). Several other SAPKs, such as ZmSAPK8, OsSAPK4, and PtSnRK2.5/2.7, act as positive regulators of multiple stress tolerances (Diédhiou et al., 2008; Ying et al., 2011; Song et al., 2016). Therefore, *SAPKs* are excellent candidate genes for stress tolerance improvement.

Perennial ryegrass (*Lolium perenne* L.) is an important cool-season perennial grass species widely cultivated in temperate regions throughout the world for its turf and forage purposes. It is important to further improve the drought stress tolerance of perennial ryegrass for better pasture and turf persistence where irrigation is often of major concern. The objectives of this study were (i) to classify and characterize *SAPKs* gene family in perennial ryegrass, and (ii) to identify *SAPKs* which can be used as candidate genes for perennial ryegrass stress tolerance.

## Materials and Methods

### Plant Materials, Growth Conditions, and Stress Treatments

Seeds of perennial ryegrass (cv. “Buena vista”) were sown in a cell tray filled with fritted clay and maintained in a greenhouse at Nanjing Agricultural University (Nanjing, China). 20 days old seedlings were transplanted and cultivated hydroponically for 2 weeks in Hoagland’s nutrient solution and maintained in a growth chamber set at a photoperiod of 14/10 h, the controlled temperature of 25/20°C (day/night), relative humidity of 70%, and photosynthetically active radiation (PAR) of 750 μmol photos m^−2^ s^−1^. The Hoagland’s nutrient solution was replaced with a fresh solution twice a week to ensure adequate oxygen and nutrient supply.

To determine the expression patterns of *LpSAPKs* involved in stress and hormone responses, plants were exposed to cold (4°C), heat (38°C), osmotic stress (15% PEG6000, w/v), NaCl (255 μm), AlCl_3_ (1.6 mm), CdCl_2_ (200 μm), 6-BA (25 μm), ABA (50 μm), and ethephon (200 μm, an ethylene releaser) treatments, respectively. Leaf and root samples were collected at 0, 0.5, 2, 6, 12, 24, & 48 h under each treatment, frozen in liquid nitrogen, and kept at −80°C for RNA extractions. Tissues including the root, crown, stem, leaf sheath, expanding leaf (the 2nd leaf from the top), mature leaf (the 3rd leaf from the top), and senescent leaf (the 4th leaf from the top) were collected from adult plants separately for RNA extractions and used for organ-specific expression analysis.

For drought tolerance assessment of perennial ryegrass lines, seeds of one commercial cultivar “Buena vista” and two selected lines bred in our research program (cv. “XiaLu-4” and “XiaLu-6,” previously named as “R2-4” and “R2-6,” respectively; Zhang et al., 2022a) were germinated on wetted paper towels with water and 10% PEG6000 in a growth chamber. 1 week later, the plant height, root length, and fresh weight of seedlings were measured, and the samples of leaf and root were collected to examine the expression level of *LpSAPK9*. Plant height was measured manually from the base to the shoot tip of each plant.

### Identification and Cloning of *LpSAPK* Genes

Coding sequence (CDS) of ten *OsSAPKs* in rice was obtained from NCBI^1^ based on the accession number reported by Zhang et al. (2018). All *LpSAPKs* gene sequences were identified using the local BLAST program to screen the perennial ryegrass genome database (Daniel et al., 2021) and our transcriptome database (Xu et al., 2019; Zhang et al., 2022b) with ten *OsSAPKs* sequences as quires. The E-value threshold for the BLAST program was set at 1e−10 to obtain the candidate dataset of *SAPK* genes. The local perennial ryegrass genome database and transcriptome databases were built using Bioedit software.^2^ Sequences of all *LpSAPKs* were examined using the Conserved Domain Database^3^ to confirm whether these proteins have typical features of the SAPKs. The biophysical properties of the *LpSAPKs*, such as Molecular weight (Mw), Isoelectric point (pI), and Grand average of hydropathy (GRAVY), were calculated using the ProtParam tool.^4^

Total mRNA was extracted from different tissues of perennial ryegrass using the Plant RNA Kit (Omega Bio-Tek, United States). The first-strand cDNA was synthesized using a cDNA Reverse Transcription Kit (TaKaRa, Dalian, China) and was used for gene cloning. All confirmed *LpSAPK* genes were cloned by using primers designed according to the sequences flanking their open reading frame (ORF; Supplementary Table S1). PCR was performed in a 50 μl reaction volume using Phanta Super-Fidelity DNA Polymerase from Vazyme (Vazyme Biotech Co., Ltd., Nanjing, China) for gene cloning. The PCR products were purified using the Gel Extraction Kit (Omega Bio-Tek, United States) and cloned into a modified pEND-linker vector for sequencing.

### Phylogenetic Tree, Genic Structure, Motif, Ka/Ks, and Promoter Analyses

A phylogenetic tree was constructed using the neighbor-joining (NJ) method in the MEGA 7.0 software program with 1,000 bootstrap replicates, p-distance model/method, uniform rates among sites, partial deletion with 50% Cutoff gaps/missing data treatment, and the number of threads is seven. We have added this information in the revised MS. In addition to *LpSAPKs* from perennial ryegrass, their orthologous proteins (SAPK/SnRK2) from Arabidopsis (*Arabidopsis thaliana*), rice (*Oryza sativa*), maize (*Zea mays*), and brachypodium (*Brachypodium distachyon*) were used for the phylogenetic analysis. Multiple amino acid sequence alignment of *LpSAPKs* was performed using online Multiple Sequence Alignment tool.^5^ The gene structures of the *LpSAPKs* were determined based on the comparison of their coding sequences (CDS) and genomic sequences. The online MEME program (version 5.4.1)^6^ was used to identify the conserved motifs of the LpSAPK proteins. The Ka/Ks analysis was performed using TBtools (Chen et al., 2020a). Two kb nucleotide sequences upstream from the transcription initiation site were considered as promoters of each gene. The *cis* elements of *LpSAPK* promoters were obtained from the PLACE database.^7^

### Subcellular Localization Analysis

The CDS of the *LpSAPKs* were amplified with gene-specific primers (Supplementary Table S1) and cloned into the pEarleyGate103 vector to generate *LpSAPK-GFP* fusion genes and transformed into *Agrobacterium tumefaciens* strain “*AGL1.”* Then, the resuspended *Agrobacterium* at OD600 = 0.6 in 1/4 MS salt solution was injected into the leaves of 4 to 6-week-old *Nicotiana benthamiana* plants. The injected leaves were incubated in the dark for 12 h and then moved to a normal growth environment for 3 days, and then examined the GFP fluorescence signal using a Zeiss LSM 800 laser scanning confocal microscope (Carl Zeiss SAS, Jena, Germany). The subcellular localizations of *LpSAPKs* were also predicted by using the online PSORT software.^8^

### qRT-PCR Analysis

For qRT-PCR analysis, the same procedure was used as reported previously (Zhang et al., 2016b, 2021). Detailed information on primers used for the qRT-PCR analysis was listed in Supplementary Table S1, and *LpeIHF4A* was selected as the reference gene (Huang et al., 2014). The relative expression levels of *LpSAPK* genes were calculated using the 2^−ΔΔCT^ method.

### Heterologous Expression of *LpSAPKs* in Yeast

Two *Saccharomyces cerevisiae* yeast mutant strains, △*hog1* (Winkler et al., 2002) and △*G19* (Quintero et al., 1996), that were sensitive to drought and salt treatments, were used to screen stress-related *LpSAPKs*. The CDS of the *LpSAPKs* were cloned into the pGAD426 vector and then transformed to the yeast strains using the Frozen-EZ Yeast Transformation Kit (Zymo Research, United States). The transformed yeast strains were grown in the synthetic dropout uracil (SD-Ura) or synthetic dropout uracil histidine (SD-Ura-His) medium. The pGAD426-*GUS* plasmid was used as the negative control. For drought tolerance assessment, 10 μl △*hog1* yeast solution (OD_600_ = 1) was spotted on solidified SD-Ura medium containing 0.75 M sorbitol and incubated at 28°C for 3 days. The growth of transformants in SD-Ura liquid medium containing 0.75 M sorbitol was determined by measuring OD_600_ at 24 h intervals. The transformed yeast mutant strain△*G19* was used for salt tolerance by assessing their growth in SD-Ura-His medium containing 500 mm NaCl.

### Statistical Analysis

Data in this study were statistically analyzed by the LSD and Duncan test program at a significance level of 0.05 using the SPSS software (Version 12, SPSS Inc., Chicago, IL). Data were expressed as means ± standard error (SE).

## Results

### Identification of *SAPK* Genes in Perennial Ryegrass

Ten *LpSAPK* genes were isolated from the perennial ryegrass genome and transcriptome databases and named as *LpSAPK1*-*10* after corresponding to their orthologous proteins in rice. Subsequently, their CDSs were cloned and sequenced (GenBank accession numbers were listed in Supplementary Table S2). The CDS of the *LpSAPKs* vary from 1,017 bp (*LpSAPK3*) to 1,143 bp (*LpSAPK9*) and their encoded proteins range from 338 aa to 380 aa with molecular weights of 38.30 kDa (LpSAPK3) to 43.52 kDa (LpSAPK9). All LpSAPKs have isoelectric points (pI) less than 7.0, indicating they are acidic hydrophilic proteins (GRAVY <0) according to the GRAVY analysis (Supplementary Table S2).

**Figure 1:**
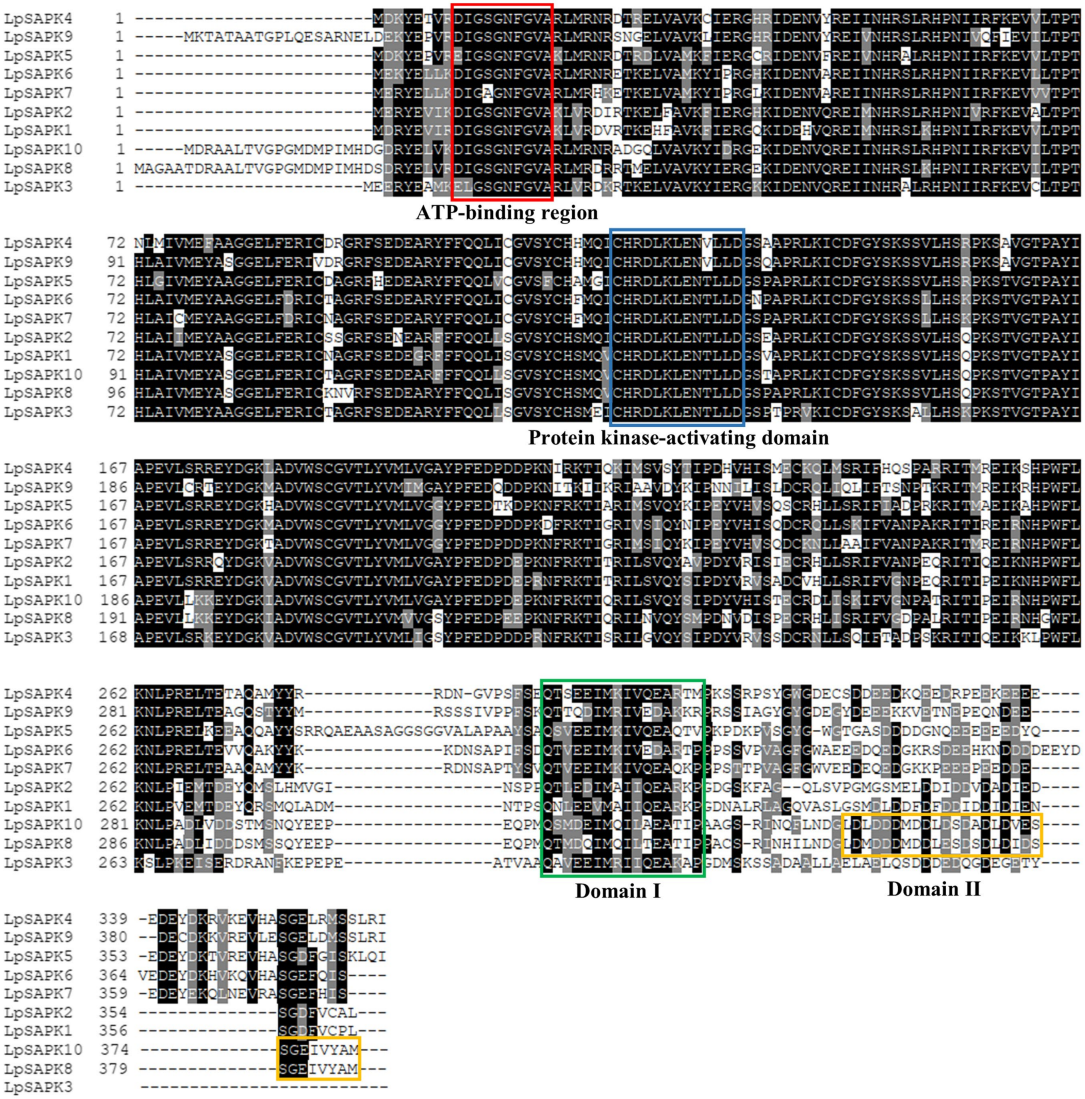
Multiple sequence alignment of *LpSAPKs*. The protein kinase ATP-binding region signature and the serine/threonine-protein kinase active-site signature were indicated by a red box and a blue box, respectively. Domain I and Domain II at the C-terminus were marked by a green box and a yellow box, respectively.

Furthermore, all *LpSAPKs* have typical domains of the SAPK family, including the ATP-binding region (DI/LGXGXFGVA) and protein kinase-activating domain (I/VCHRDLKLENTLLD) in N-terminal regions that constitute the serine/threonine kinase domain, and the domain I and domain II in the C-terminal regions (Figure 1). It is interesting to note that domain I is present in all LpSAPKs, while domain II is specific to LpSAPK8 and LpSAPK10 (Figure 1).

### Phylogenetic Analysis, Conserved Domain, and Motifs of LpSAPKs

**Figure 2:**
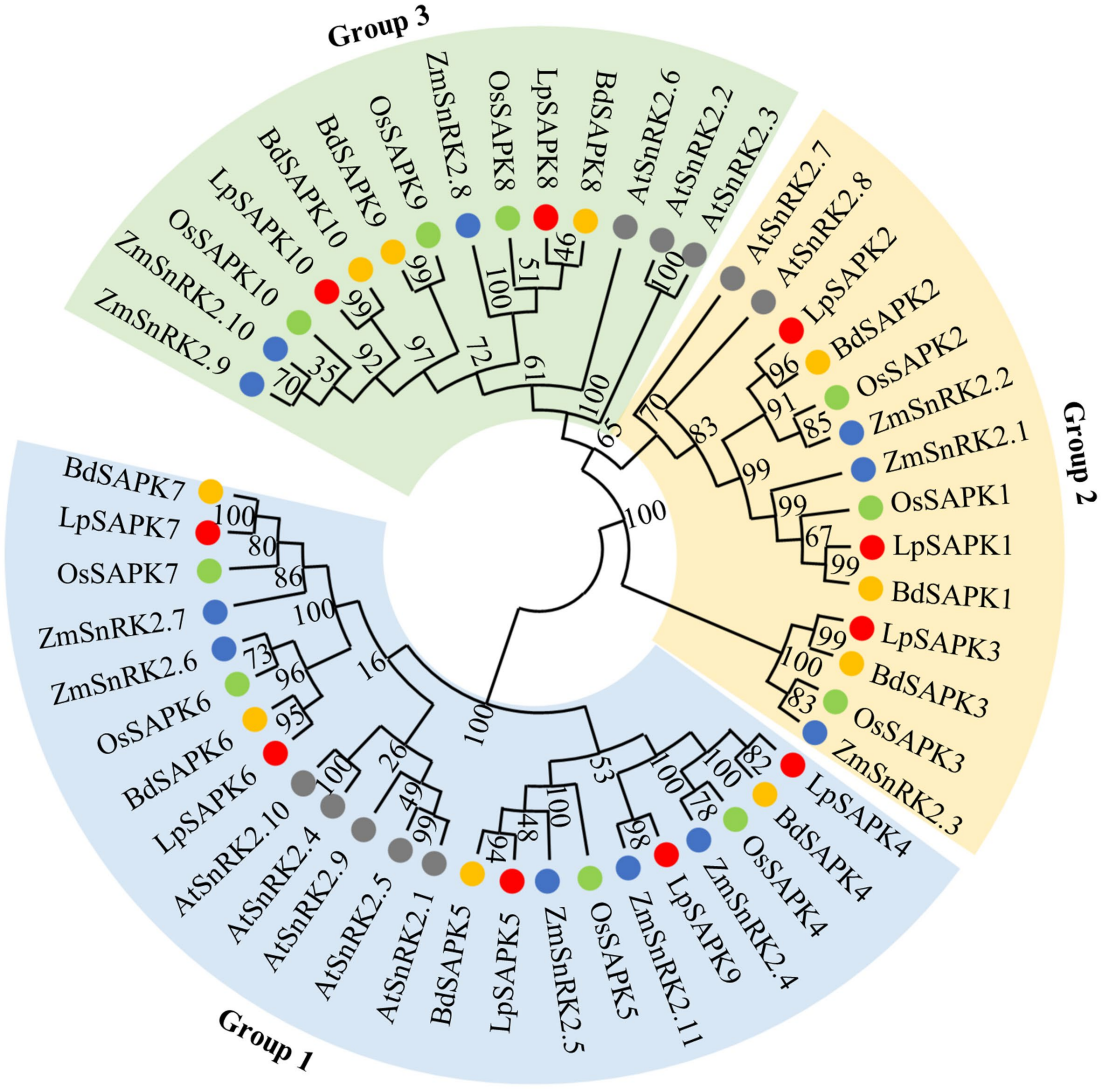
Phylogenetic analysis of SAPK/SnRK2 proteins from Arabidopsis (AtSnRK2s), rice (OsSAPKs), and maize (ZmSnRK2s), brachypodium (BdSAPKs), and perennial ryegrass (*LpSAPKs*). The phylogenetic tree was constructed using the neighbor-joining (NJ) method with 1,000 bootstrap replicates in MEGA7.0. The Genebank accession numbers of *LpSAPK*, AtSnRK2, OsSAPK2, ZmSnRK2, and BdSAPK proteins are listed in Supplementary Tables S2, S3.

To illustrate the evolutionary relationship among SAPK/SnRK2 homologs in different plants, a phylogenetic tree was constructed comprising 10 *LpSAPKs* in perennial ryegrass, 10 OsSAPKs in rice, 11 ZmSnRK2s in maize, 10 BdSAPKs in brachypodium, and 10 AtSnRK2s in Arabidopsis (Figure 2; Supplementary Table S3). The SAPKs were clustered into three subclasses, namely, groups 1, 2, and 3 (Figure 2). Group 1 comprised 5 LpSAPKs, including LpSAPK4/5/6/7/9; the group 2 comprised 3 ones, namely, LpSAPK1/2/3; while the rest two (LpSAPK8/10) were classified in the group 3.

**Figure 3:**
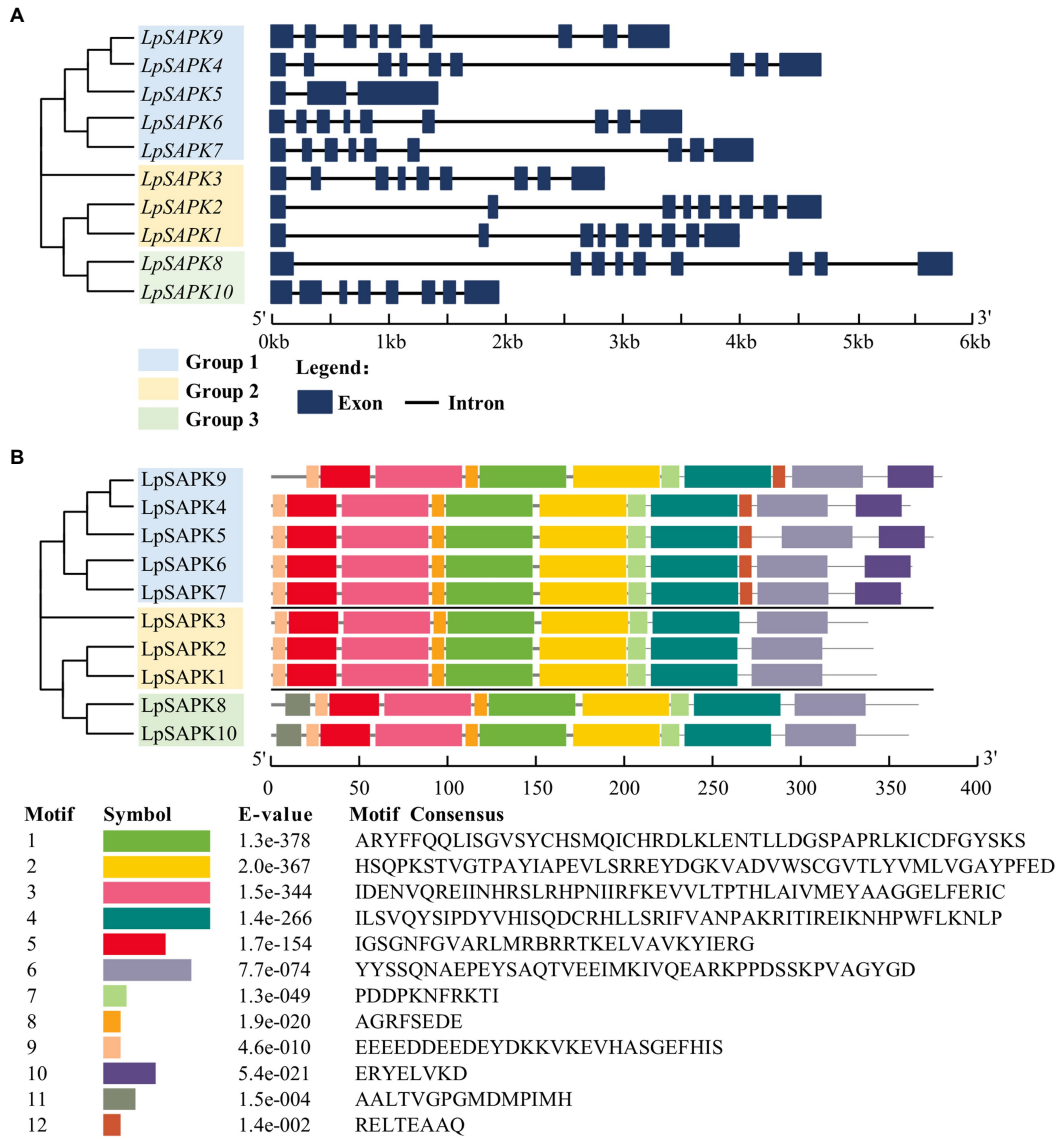
Phylogenetic relationships, genic structures, and conserved motif composition of *LpSAPKs*. **(A)** Phylogenetic relationships and exon–intron organizations of the *LpSAPKs*. Exons and introns are shown by filled boxes and single lines, respectively. **(B)** Motif analysis of *LpSAPKs*. Each colored box represents a motif in the protein, with the motif name indicated at the bottom of the figure.

Most *LpSAPKs* exhibit similar exon–intron organizations that *LpSAPK1*/*2/3/4/6/7/8/9* have 9 exons, *LpSAPK10* has 8 exons, while *LpSAPK5* only has 3 exons (Figure 3A). A total of 12 conserved motifs were identified by MEME analysis (Figure 3B). The motifs 1–5 and 7–9 were found in all LpSAPK proteins, while the motif 11 was specific to the group 3 LpSAPKs, and motifs 10 & 12 were specific to the group 1 LpSAPKs. Moreover, the identified motifs 1, 4, and 5 were the protein kinase-activating domain, the domain I, and the ATP-binding region, respectively.

### *LpSAPKs* Were Under Purifying Selection

Since the phylogenetic classification and genic structures of *LpSAPKs* were similar to those in other plant species, we speculated that the *LpSAPKs* might be under purifying selection during evolution to maintain their conserved functions. To verify this hypothesis, we performed the Ka/Ks analysis using *LpSAPKs* and their close orthologous genes in rice and brachypodium. As shown in Table 1 all ten *LpSAPKs* and their compared orthologs had ka/ks ≦ 0.2, suggesting that all of these genes were under purifying selection.

**Table 1:** Purifying selection of *LpSAPKs* and their orthologous genes in rice (*OsSAPKs*) and brachypodium (*BdSAPKs*).

Gene 1	Gene 2	Ka	Ks	Ka/Ks	Evolutionary selection
*LpSAPK1*	*OsSAPK1*	0.032	0.596	0.053	Purifying
*LpSAPK2*	*OsSAPK2*	0.055	0.677	0.081	Purifying
*LpSAPK3*	*OsSAPK3*	0.063	0.592	0.106	Purifying
*LpSAPK4*	*OsSAPK4*	0.053	0.483	0.110	Purifying
*LpSAPK5*	*OsSAPK5*	0.097	0.731	0.133	Purifying
*LpSAPK6*	*OsSAPK6*	0.063	0.669	0.095	Purifying
*LpSAPK7*	*OsSAPK7*	0.043	0.468	0.092	Purifying
*LpSAPK8*	*OsSAPK8*	0.021	0.514	0.041	Purifying
*LpSAPK9*	*OsSAPK4*	0.144	0.736	0.195	Purifying
*LpSAPK10*	*OsSAPK10*	0.025	0.519	0.048	Purifying
*LpSAPK1*	*BdSAPK1*	0.015	0.419	0.036	Purifying
*LpSAPK2*	*BdSAPK2*	0.030	0.431	0.071	Purifying
*LpSAPK3*	*BdSAPK3*	0.046	0.457	0.101	Purifying
*LpSAPK4*	*BdSAPK4*	0.041	0.309	0.133	Purifying
*LpSAPK5*	*BdSAPK5*	0.073	0.571	0.128	Purifying
*LpSAPK6*	*BdSAPK6*	0.029	0.358	0.082	Purifying
*LpSAPK7*	*BdSAPK7*	0.021	0.245	0.084	Purifying
*LpSAPK8*	*BdSAPK8*	0.014	0.246	0.058	Purifying
*LpSAPK9*	*BdSAPK4*	0.155	0.756	0.205	Purifying
*LpSAPK10*	*BdSAPK10*	0.008	0.359	0.024	Purifying

### Subcellular Localization of LpSAPK Proteins

**Figure 4:**
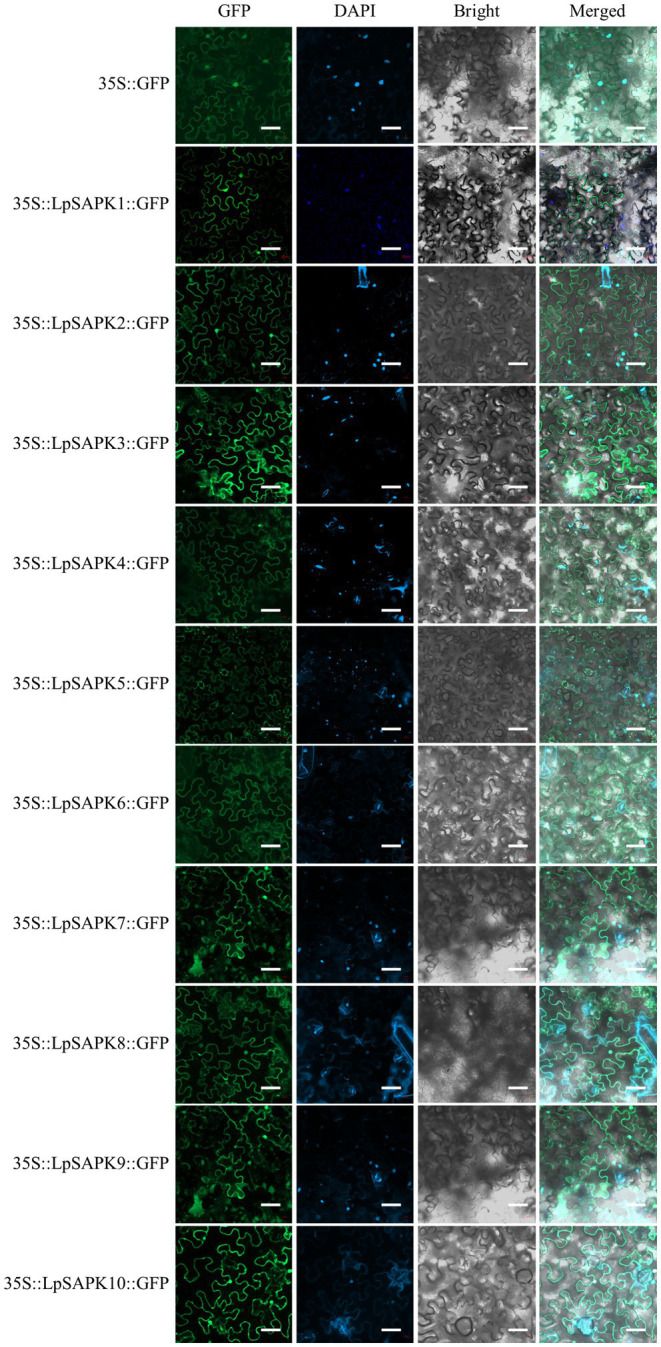
Subcellular localization of the LpSAPK proteins. The vector *35S*::*GFP* was used as the control. All *LpSAPKs* were infusion with a GFP tag and visualized as green fluorescence in leaf cells of *Nicotiana benthamiana*.

Using the PSORT software, we predicted that all *LpSAPKs* had relatively high affirmatives to the nucleus and cytosol (Supplementary Table S4). Consistently, we observed that the subcellular localization of LpSAPKs-GFP was in both the cytoplasm and the nucleus of leaf epidermal cells, while the only exception went for LpSAPK5 which was mainly detected in the cytoplasm of guard cells but not in the nucleus (Figure 4).

### *Cis* Elements in *LpSAPKs*’ Promoters

Promoters of all *LpSAPK*s were amplified from perennial ryegrass and sequenced for *cis* elements analysis. As shown in Table 2, these *LpSAPK*s promoters have multiple *cis* elements involved in stress and hormone signaling pathways. For example, the ABRE (MACGYGB) and the CGTCA-motif were found in all *LpSAPKs* promoters. The ABRE *cis* element was the core DNA-binding sequence by ABI3 and ABF4 that were key transcription factors in the ABA signaling pathway. Similarly, the CGTCA-motif was one major *cis*-acting regulatory element involved in the MeJA (methyl jasmonate) pathway and was also identified in all *LpSAPK* promoters. The ethylene-responsive element (ERE) was found in the promoters of *LpSAPK3/4/5/6/7/9*. The gibberellin-responsive (GARE) was present in the promoters of *LpSAPK1/2/7/9*. The TGA element, involved in the auxin signaling pathway, was present in the promoters of *LpSAPK5/6/7/8/9*. The MYB binding site was found in the promoters of *LpSAPK5/6/7/9*. The TC-rich repeat, a defense and stress-responsive element, was found in the promoters of *LpSAPK6/8/9*. And the low-temperature responsive element (LTR) was identified in the promoters of *LpSAPK5/7* (Table 2).

**Table 2:** The *cis* element analysis of *LpSAPKs* promoter regions.

*cis* element	Functional annotation	*LpSAPK* genes									
		1	2	3	4	5	6	7	8	9	10
ABRE	Abscisic acid responsiveness	3	5	6	1	4	3	2	5	5	2
CGTCA	MeJA-responsiveness	4	2	3	4	8	5	6	1	4	3
ERE	ethylene-responsive element	0	0	1	1	1	1	1	0	2	0
GARE	gibberellin-responsive	1	1	0	0	0	0	3	0	2	0
TGA element	auxin-responsive element	0	0	0	0	2	1	2	4	2	0
TCA element	salicylic acid responsiveness	0	1	0	0	0	2	0	2	1	0
LTR	low-temperature responsiveness	0	0	0	0	1	0	2	0	0	0
MBS	MYB binding site involved in drought-inducibility	0	0	0	0	2	1	2	0	1	0
TC-rich repeats	defense and stress responsiveness	0	0	0	0	0	1	0	1	2	0

### Expression Profiles of *LpSAPKs* in Different Tissues and in Response to Abiotic Stress and Hormone Treatments

**Figure 5:**
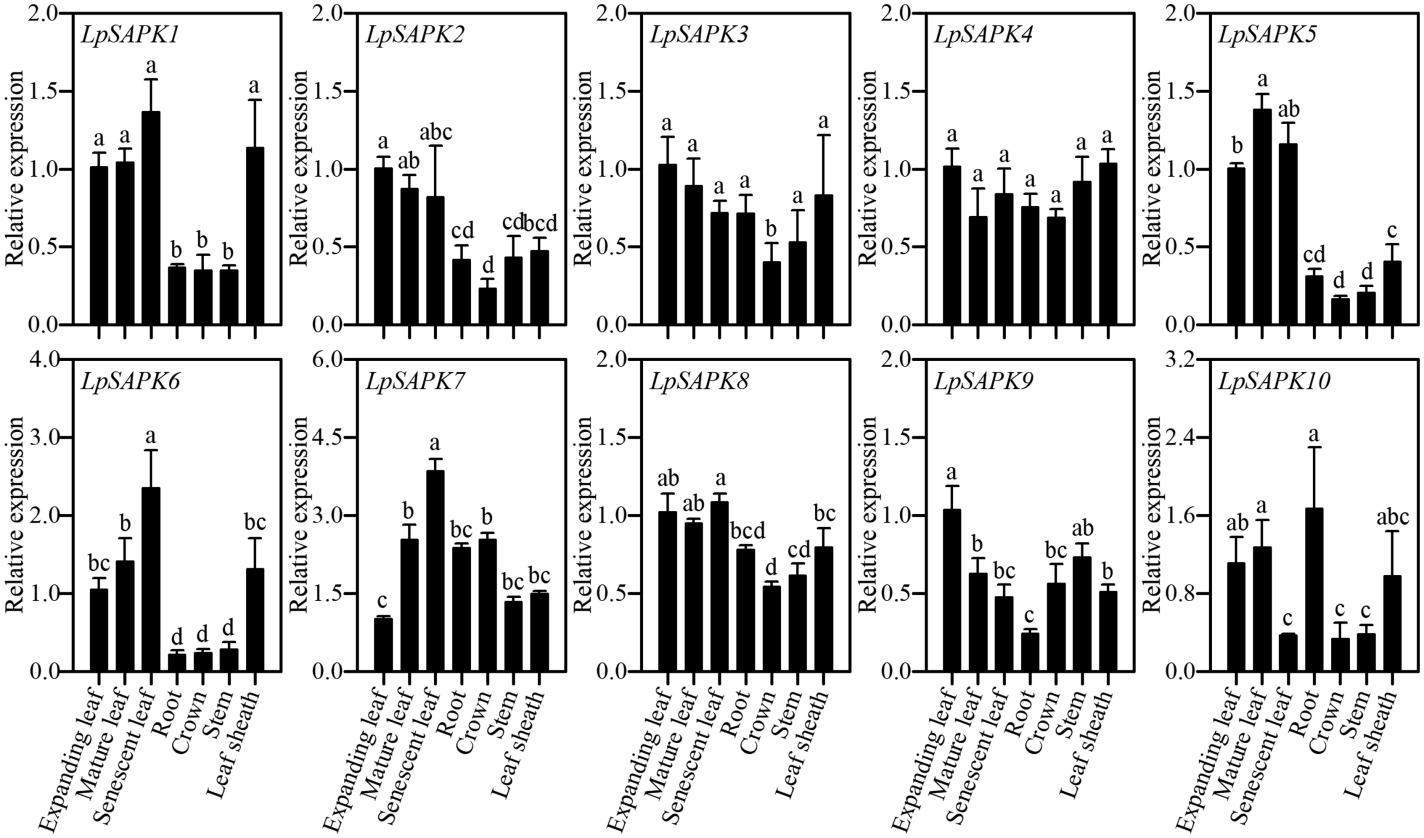
Relative expression levels of *LpSAPKs* in different organs. Data are means ± SE (*n* = 4). Different letters above bars represent significant differences at *p* ≤ 0.05.

Expression patterns of 10 *LpSAPKs* were analyzed in seven tissues, including root, crown, stem, leaf sheath, expanding, mature and senescent leaves. As shown in Figure 5, five *LpSAPK*s (*LpSAPK1*/*2/5/6/8*) had similar expression patterns that were higher in leaves than in stem, crown, and root; expression levels of *LpSAPK6* and *LpSAPK7* were gradually and significantly increased along with leaf aging, while the contrary was true for *LpSAPK9*; and *LpSAPK3*&*4* showed similar expression levels in all tested tissues.

**Figure 6:**
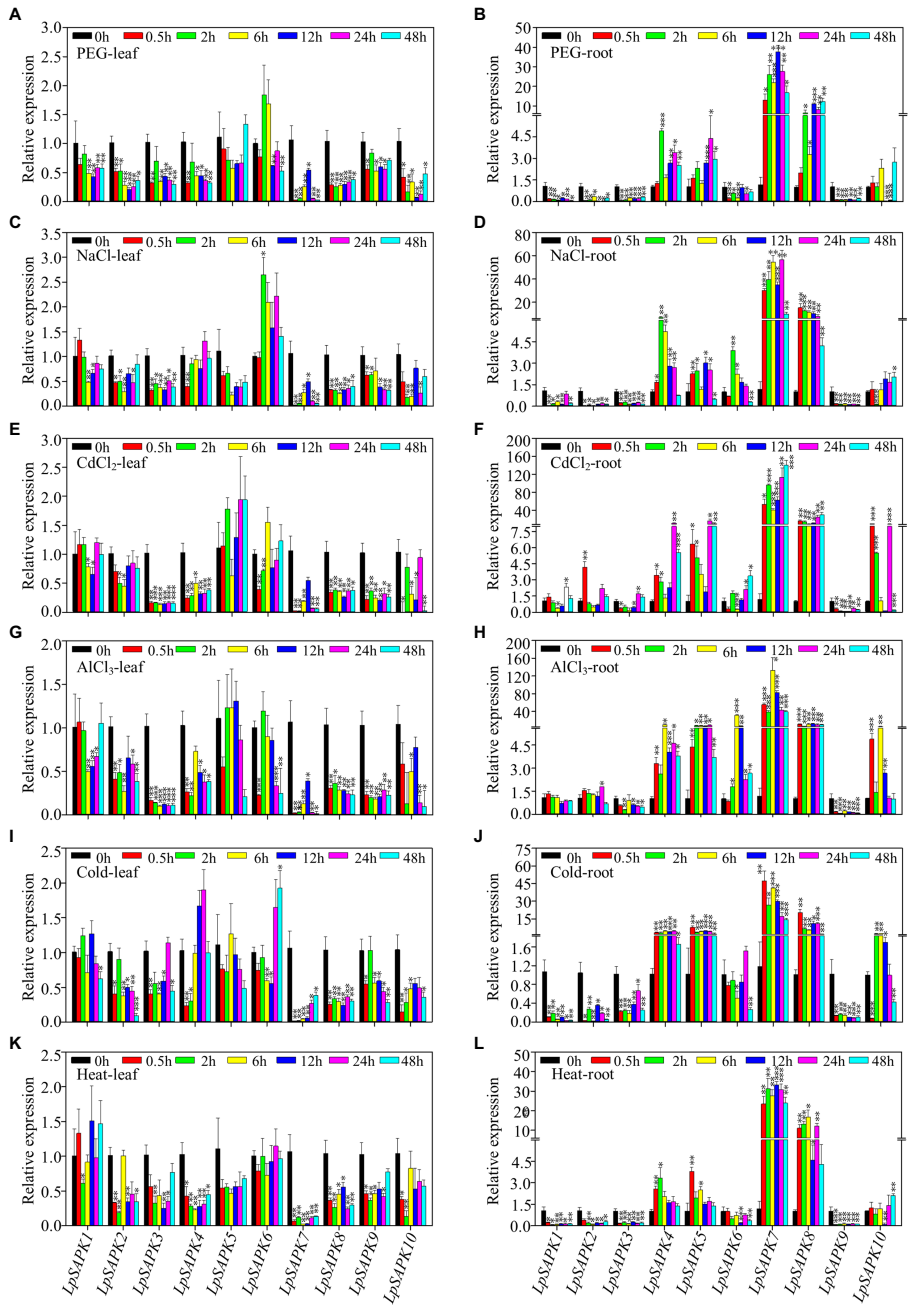
Relative expression levels of *LpSAPKs* in leaves and roots under six different abiotic stresses. **(A,B)** osmotic stress with 15% PEG6000; **(C,D)** salt stress; **(E–H)** heavy mental stress (200 μm CdCl_2_ and 1.6 Mm AlCl_3_); **(I,J)** cold stress (4°C); **(K,L)** heat stress (38°C). Data are means ± SE (*n* = 4). ^*^, ^**^, and ^***^ above the bars represents significant difference compared to the values at 0 h at p ≤ 0.05, 0.01, and 0.001, respectively.

Then, relative expression levels of *LpSAPKs* were analyzed under six different abiotic stress conditions (PEG, NaCl, CdCl_2_, AlCl_3_, cold, and heat) at seven-time points. In general, these six stress treatments led to suppressed expression of eight *LpSAPKs* (except for *LpSAPK5* and *LpSAPK6*) in leaves, and suppressed expression of *LpSAPK1*/*2*/*3*/*9* but upregulated expression of *LpSAPK4*/*5*/*7*/*8* in roots over two-fold changes (Figure 6). It is also notable that the transcriptional responses of these *LpSAPKs* to these stress treatments were more dramatic in roots than in leaves. For example, the expression levels of *LpSAPK7/8* increased by 10- to 120-fold in roots but decreased by less than 20 folds in leaves in response to different abiotic stress (Figure 6).

**Figure 7:**
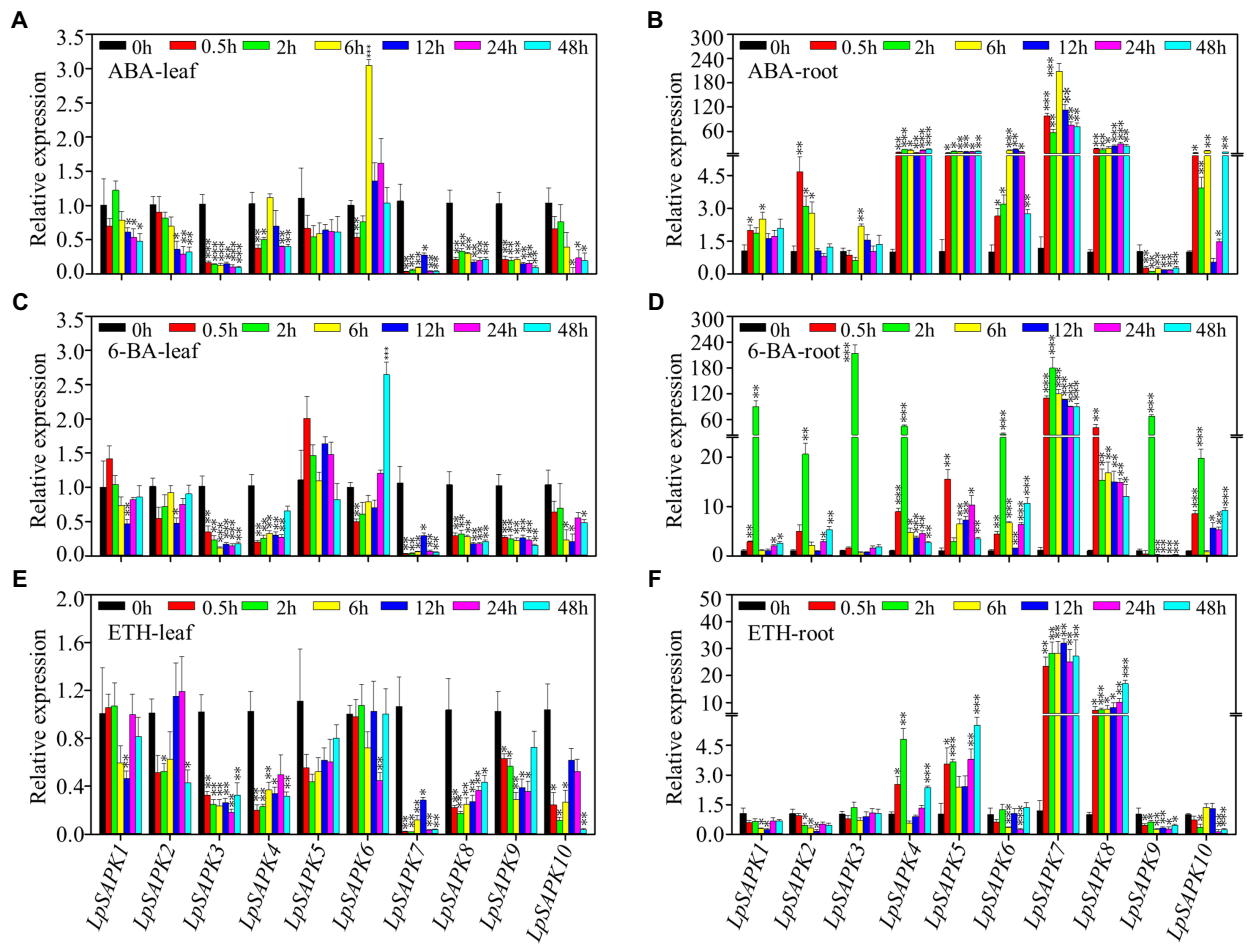
Relative expression levels of *LpSAPK*s in leaves and roots under three hormone treatments. **(A,B)** ABA treatment; **(C,D)** cytokinin (6-BA) treatment; **(E,F)** ethephon treatment. Data are means ± SE (*n* = 4). ^*^, ^**^, and ^***^ above the bars represent significant differences compared to 0 h at *p* ≤ 0.05, 0.01, and 0.001, respectively.

We also analyzed the relative expression levels of *LpSAPKs* in response to ABA, cytokinin (6-BA), and ethephon (ETH) treatments. As shown in Figure 7, nine out of ten *LpSAPKs* (except *LpSAPK5*) were responsive to ABA treatment, among which eight were downregulated but the *LpSAPK6* was upregulated in leaves. The contrary trend was observed in roots that nine *LpSAPKs* (except *LpSAPK9*) were upregulated by ABA treatments. In response to 6-BA treatment, expression levels of *LpSAPK3*/*4*/*7*/*8*/*9* were downregulated in leaves, while nine *LpSAPKs* (except *LpSAPK9*) were upregulated by 6-BA in roots. Under ethephon treatment, nine *LpSAPKs* (except *LpSAPK5*) were downregulated in leaves; while in roots, *LpSAPK1*/*2*/*6*/*9*/*10* were suppressed, and *LpSAPK4/5*/*7*/*8* were upregulated (Figure 7).

### Identification of *LpSAPK9* as a Potential Positive Regulator in Drought Stress

**Figure 8:**
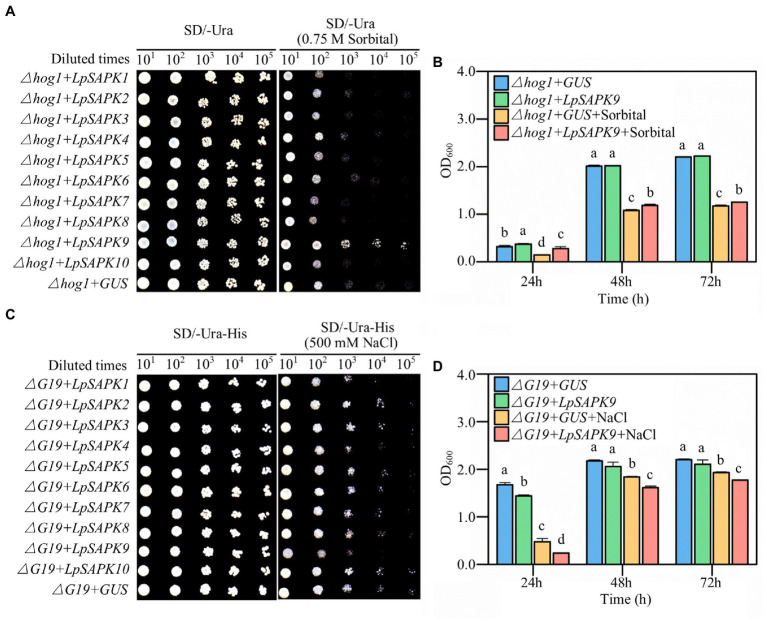
Drought and salt tolerance assessment of yeast cells with heterologous overexpressed of *LpSAPKs*. **(A,B)** The growth of ∆hog1 yeast mutants transformed with the *GUS* gene (negative control) or with all the *LpSAPKs* on SD-Ura medium with or without sorbitol. **(C,D)** The growth of ∆G19 yeast mutants transformed with the *GUS* gene or with all the *LpSAPKs* on SD-Ura-His medium with or without NaCl. Data in b and c are means ± SE (*n* = 4). Different letters above bars represent significant differences at p ≤ 0.05.

To screen potential *LpSAPKs* regulating drought and salt tolerance, we expressed each of these genes in two yeast mutant strains that were susceptible to drought or salt stress. As shown in Figure 8, the ∆*hog1* cells harboring *LpSAPK9* exhibited remarkably enhanced growth when compared with the *GUS* control on the SD-Ura agar medium with 0.75 M sorbitol (Figure 8A). Quantitatively, the *LpSAPK9/*∆*hog1* lines grew at significantly higher rates than the *GUS/*∆*hog1* control lines after 24, 48, and 72 h of incubation in SD-Ura liquid medium with 0.75 M sorbitol (Figure 8B). Yet, *LpSAPK9/*∆*G19* lines grew at significantly slower rates than the *GUS/*∆*G19* control lines when the medium was supplemented with 500 mm NaCl (Figures 8C,D). For the rest *LpSAPK* genes, no significant effect was observed on drought or salt tolerance of the yeast mutants.

**Figure 9:**
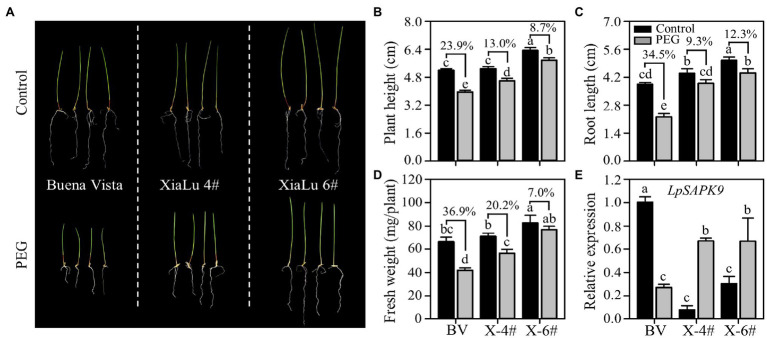
Expression of *LpSAPK9* to drought stress in three perennial ryegrass cultivars differing in drought tolerance. **(A)** The phenotype of three perennial ryegrass cultivars under control and drought stress conditions. **(B–D)** Growth traits (e.g., plant height, root length, and above-ground biomass) of three perennial ryegrass cultivars under control and drought stress conditions. “BV” and “X” in **(D)** represent “Buena Vista” and “XiaLu.” **(E)** Expression levels of *LpSAPK9* of three perennial ryegrass cultivars under control and drought stress conditions. Data are means ± SE (*n* = 4). Different letters above bars represent significant differences at p ≤ 0.05.

To further verify whether *LpSAPK9* was involved in plant drought tolerance, we measured its expression levels in three varieties of perennial ryegrass differing in drought tolerance. Notably, “Xialu-4” and “XiaLu-6” bred in our own program had exceptional drought tolerance. Consistently, the PEG-induced growth inhibitions on “XiaLu-4” and “XiaLu-6” were significantly lower than that in “Buena vista” with less decreased plant height, root length, and fresh weight (Figures 9A–D). Under the control condition, the expression level of *LpSAPK9* in “Buena vista” was higher than those in “XiaLu-4” and “XiaLu-6”; yet under PEG treatment, the expression of *LpSAPK9* decreased in “Buena vista” but was significantly increased in “XiaLu-4” and “Xialu-6” (Figure 9E).

## Discussion

Several *SAPKs/SnRK2s* were identified as important regulators in plant growth and development, ABA signaling transduction, and stress tolerance (Kobayashi et al., 2004; Diédhiou et al., 2008; Huai et al., 2008; Bai et al., 2017). Yet, the *SAPK* family gene(s) has not been characterized in perennial ryegrass before. In this study, we identified and cloned 10 *LpSAPKs* in perennial ryegrass using the transcriptomic and genomic databases, characterized expression patterns of *LpSAPKs*, and screened out a potential positive regulator (*LpSAPK9*) in plant drought tolerance.

The number of *SAPKs* proteins in perennial ryegrass was similar to other plant species, such as Arabidopsis (10 *SAPKs/SnRK2s*; Boudsocq et al., 2004), rice (10; Kobayashi et al., 2004), maize (11; Huai et al., 2008), and brachypodium (10; Wang et al., 2015), in spite of the big variation of their genome sizes. The phylogenetic trees of LpSAPKs in ryegrass, ZmSnRK2s in maize, OsSAPKs in rice, and AtSnRK2s in Arabidopsis were highly similar in three subgroups (Boudsocq et al., 2004; Kobayashi et al., 2004; Huai et al., 2008). And all *SAPKs*/*SnRK2s* in cotton, Arabidopsis, and rice were found to have similar genic structures with nine exons (Boudsocq et al., 2004; Huai et al., 2008; Liu et al., 2017). In *LpSAPKs*, eight have nine exons with two exceptions (*LpSAPK5* and *LpSAPK10*). The coding sequenced-based phylogenetic analysis and full genic sequence-based gene structural (exon–intron) analysis revealed that the *SAPKs* was a conserved family across dicot and monocot species. Furthermore, the Ka/Ks analysis showed that all *LpSAPKs* and their orthologs in rice and brachypodium were less than 0.205, suggesting that these genes were under strong purifying selection to keep their conserved functions across different plant species. During evolution, fixation of advantageous mutations would lead to evolutionary innovations causing plants’ divergence and plants’ adaptation to new or constantly changing environments; while removal of deleterious mutations was essential for plants to maintain their “essential” functions (Paterson et al., 2004). Purifying selection of all *LpSAPKs* suggested that these genes were essential or even indispensable for plants’ survival in nature and their orthologous genes should have similar functions across different plant species.

The C-terminal domain of SnRKs consisted of two subdomains, Domain I and Domain II. Since Domain II was only found in a few specific SnRK2s, it might be responsible for their functional diversity, for example, through interaction with PP2Cs for ABA signaling transduction (Kobayashi et al., 2004; Fujita et al., 2013). In the present study, all the *LpSAPKs* consist of Domain I, while only LpSAPK8 and LpSAPK10 harbor Domain II. At the transcriptional level, LpSAPK8/10 also responded to ABA treatments. Together, this result indicated that LpSAPK8&10 were likely involved in ABA signaling transduction.

*LpSARKs* are responsive to hormones and various abiotic stress treatments and are often in the opposite trend in roots and in leaves (Figures 7, 8). Similar results were also found in other plant species (Boudsocq et al., 2004, 2007; Kobayashi et al., 2004; Fujita et al., 2009). For example, most Arabidopsis *SnRK2s* (except *AtSnRK2.9*) were responsive to sucrose, mannitol, sorbitol, and NaCl treatments; *AtSnRK2.2*/*2.3*/*2.6*/*2.*7/*2.8* were responsive to ABA treatment; but no *AtSnRK2* gene was responsive to low temperature (Boudsocq et al., 2004). Notably, nearly all *LpSAPKs* responded to stress and hormone treatments in contrasting trends at the transcriptional level. For example, *LpSAPK5* was not responsive to all tested treatments in leaves but was upregulated by all tested treatments in roots. This contrasting result for *LpSARKs* in leaves and roots suggested that these protein kinases should be involved in plant stress-inducible development alternations. For example, under mild drought conditions, plants could develop deeper root systems with reduced leaf sizes. It would be interesting to carry out more functional tests on these *LpSAPKs* in the future.

On the other hand, the promoter analysis of these genes revealed a number of *cis* elements associated with hormone and stress responses, such as the ABA-response element (ABRE), gibberellin-response element (GARE), ethylene-responsive elements (ERE), methyl jasmonate response element (CGTCA), low-temperature stress response element (LTRE), stress response element (TC-rich repeats), and MYB binding site for drought-inducible element (MBS). Distinct distributions of *cis* elements were found on promoters of *LpSAPKs*. For example, no ERE was identified in the promoters of *LpSAPK1/2*/*/10*, although these genes were responsive to the ethephon treatment. And no MBS and LTR element was found on promoters of *LpSAPK1*/*2*/*3/4/8/10* although these genes were responsive to drought and cold stress treatments. Similar results were also found in wheat (Zhang et al., 2016a), tea (Zhang et al., 2018), and soybean (Zhao et al., 2017). These findings indicated that there were likely other unrecognized stress-related *cis* elements or post-transcriptional regulation (e.g., by small RNAs) involved in the regulation of *LpSAPKs*.

Several functionally characterized SAPKs/SnRK2s were known for their regulatory roles in plant abiotic stress tolerance. For example, overexpression of *AtSnRK2s*, *OsSAPKs*, or *TaSnRK2s* enhanced plant tolerance to abiotic stresses (Diédhiou et al., 2008; Umezawa et al., 2009; Mao et al., 2010; Zhang et al., 2011). In order to quickly assess their roles in stress tolerance, a yeast heterologous expression system was used. It was found that heterologous expression of the *LpSAPK9* increased drought tolerance, but decreased salt tolerance to yeast. However, heterologous expression of the rest *LpSAPKs* showed no obvious effect on drought or salt stress tolerance in yeast. Furthermore, the expression of *LpSAPK9* in different ryegrass cultivars differing in their drought tolerance showed contrasting changes, that is, decreased in leaves of the drought-sensitive variety but increased in the more drought-tolerant varieties. These contrasting expression changes of *LpSAPK9* coincided with the drought tolerance degrees of the varieties, suggesting that this gene might be one important regulator in ryegrass drought tolerance. On the other hand, this diversified expression pattern of *LpSAPK9* in different ryegrass varieties also reiterated the possibility that this *SAPK* gene(s) could have been selected and used in ryegrass domestication and breeding.

## Conclusion

In the present study, the *SAPK/SnRK2* genes were identified and characterized using both *in silico* and experimental approaches in perennial ryegrass. The *LpSAPKs* were under strong purifying selection that showed similar genic structures and phylogenetic relationships to their orthologous genes in closely related species. Yet, the promoter regions of these *LpSAPKs* as well as their expression patterns were different from their orthologs, suggesting that even though their functions were conserved but still could have diverged at the transcriptional level. Furthermore, *LpSAPK9* was screened out as a potential positive regulator in ryegrass drought tolerance. Our results will facilitate further functional analysis of *LpSAPK* family genes for molecular breeding of ryegrass.

## Data Availability Statement

The datasets presented in this study can be found in online repositories. The names of the repository/repositories and accession number(s) can be found in the article/Supplementary Material.

## Author Contributions

JZ and BX conceived the project. JX, RZ, QZ, XH, and TY performed the experiments and analyzed the data. JX and JZ wrote the paper. BX revised the manuscript. All authors contributed to the article and approved the submitted version.

## Funding

This work was supported by Jiangsu Agriculture Science and Technology Innovation Fund [grant no. CX(21)3004] and National Natural Science Foundation of China (Grant no. 31971757).

## Conflict of Interest

The authors declare that the research was conducted in the absence of any commercial or financial relationships that could be construed as a potential conflict of interest.

## Publisher’s Note

All claims expressed in this article are solely those of the authors and do not necessarily represent those of their affiliated organizations, or those of the publisher, the editors and the reviewers. Any product that may be evaluated in this article, or claim that may be made by its manufacturer, is not guaranteed or endorsed by the publisher.

## Footnotes

^1^ https://www.ncbi.nlm.nih.gov/

^2^ https://bioedit.software.informer.com/

^3^ http://www.ncbi.nlm.nih.gov/Structure/cdd/wrpsb.cgi

^4^ https://web.expasy.org/protparam/

^5^ https://www.genome.jp/tools-bin/clustalw

^6^ http://meme-suite.org/tools/meme

^7^ https://www.dna.affrc.go.jp/PLACE/

^8^ https://www.genscript.com/psort.html
