# Supplementary material for: Genome-Wide Identification and Characterization of the LpSAPK Family Genes in Perennial Ryegrass Highlight LpSAPK9 as an Active Regulator of Drought Stress
Source: Front Plant Sci. 2022 Jun 2;13:922564. doi: 10.3389/fpls.2022.922564 (PMC9201779; doi:10.3389/fpls.2022.922564)
Supplement: Supplementary file 1 [file Data_Sheet_1.docx]

**Supplementary table 1. Primers used in this study**

| Primer name | Primer Sequences (5'-3') |
| --- | --- |
| qRT-*LpSAPK1*-F | AAGGCTCGCCTCCCGAGTTA |
| qRT-*LpSAPK1*-R | GATCTCTCCTCACGCACGCC |
| qRT-*LpSAPK2*-F | GCCCCTAGCTCAGCTGCTTC |
| qRT-*LpSAPK2*-R | GATGTCCCGGACGAGCTTGG |
| qRT-*LpSAPK3*-F | CTGTCTCCTGGGCTACACGC |
| qRT-*LpSAPK3*-R | GAACTCCGATCGACAGCGGG |
| qRT-*LpSAPK4*-F | AACTGCAGCACTTGGTGTGG |
| qRT-*LpSAPK4*-R | CCAGACCACTGAAGTCGCCA |
| qRT-*LpSAPK5*-F | GCCGTCCAGCTGCCATTACT |
| qRT-*LpSAPK5*-R | GACTGTGAGGCCTACCCTGC |
| qRT-*LpSAPK6*-F | TGGGGGTGCTGTTTGTTTCGT |
| qRT-*LpSAPK6*-R | CGCTGATTTTTGCTGATGTGTGC |
| qRT-*LpSAPK7*-F | GGCCTGAGAAGACCGCAGAC |
| qRT-*LpSAPK7*-R | CAGCGACGGAACCAGTTTGG |
| qRT-*LpSAPK8*-F | TTCCCTGGAGAAGCACCCTT |
| qRT-*LpSAPK8*-R | TGGCAGAGCAGCCCAAACAA |
| qRT-*LpSAPK9*-F | TTTGTGAGGTGGGACCGTGG |
| qRT-*LpSAPK9*-R | AACTGCCTCTGCACCAGCAT |
| qRT-*LpSAPK10*-F | ACGCGACAGAAAAGGGGAGC |
| qRT-*LpSAPK10*-R | TAGCGCAGCTCTTGGTGAGC |
| CDS-*LpSAPK1*-F | ATGTGGAATTCATGGATCGGTACGAGGTGATAA |
| CDS-*LpSAPK1*-R | ATGTGACTAGTCAACGGGCACACGAAATCT |
| CDS-*LpSAPK2*-F | ATGTGGAATTCATGGAGCGGTACGAGGTG |
| CDS-*LpSAPK2*-R | ATGTGACTAGTCAACGCGCAAACGAAGTC |
| CDS-*LpSAPK3*-F | ATGTGGAATTCATGGAGGAGAGGTACGAGG |
| CDS-*LpSAPK3*-R | ATGTGACTAGTGTAGGTCTCCCCCTCGT |
| CDS-*LpSAPK4*-F | ATGTGGAATTCATGGACAAGTACGAGACGGT |
| CDS-*LpSAPK4*-R | ATGTGACTAGTTATGCGTAGCGAGCTCATG |
| CDS-*LpSAPK5*-F | ATGTGGAATTCATGGACAAGTACGAGCCTGT |
| CDS-*LpSAPK5*-R | ATGTGACTAGTGATTTGGAGCTTGCTAATGCCAAA |
| CDS-*LpSAPK6*-F | ATGTGGGATCCATGGAGAAGTACGAGCTCCT |
| CDS-*LpSAPK6*-R | ATGTGGAATTCGCTGATCTGAAACTCTCCACTAG |
| CDS-*LpSAPK7*-F | ATGTGGAATTCATGGAGAGGTACGAGCTGC |
| CDS-*LpSAPK7*-R | ATGTGACTAGTGCTGATGTGGAACTCACCG |
| CDS-*LpSAPK8*-F | ATGTGGAATTCATGGCGGGGGCGGCCACG |
| CDS-*LpSAPK8*-R | ATGTGACTAGTCATCGCATACACAATCTCTCCACT |
| CDS-*LpSAPK9*-F | ATGTGGGATCCATGAAGACGGCGACGGC |
| CDS-*LpSAPK9*-R | ATGTGGAATTCGATGCGCAGCGAGCTCATAT |
| CDS-*LpSAPK10*-F | ATGTGGAATTCATGGACCGGGCGGCGCTG |
| CDS-*LpSAPK10*-R | ATGTGACTAGTCATAGCGTACACTATCTCCCC |

**Supplementary Table 2. Information about the *LpSAPK* genes and their putative proteins.**

| Number | Gene Name | Gene ID | CDS  (bp) | Protein | | | |
| --- | --- | --- | --- | --- | --- | --- | --- |
|  |  |  |  | Length (aa) | MW (kDa) | PI | GRAVY |
| 1 | *LpSAPK1* | MZ345651 | 1032 | 343 | 39.07 | 5.36 | -0.291 |
| 2 | *LpSAPK2* | MZ345652 | 1026 | 341 | 38.69 | 5.64 | -0.242 |
| 3 | *LpSAPK3* | MZ345654 | 1017 | 338 | 38.30 | 5.49 | -0.444 |
| 4 | *LpSAPK4* | MZ345656 | 1089 | 362 | 42.12 | 6.01 | -0.648 |
| 5 | *LpSAPK5* | MZ345660 | 1128 | 375 | 42.40 | 6.10 | -0.528 |
| 6 | *LpSAPK6* | MZ345655 | 1092 | 363 | 41.82 | 5.63 | -0.561 |
| 7 | *LpSAPK7* | MZ345659 | 1074 | 357 | 40.96 | 5.69 | -0.483 |
| 8 | *LpSAPK8* | MZ345657 | 1101 | 366 | 41.51 | 4.86 | -0.324 |
| 9 | *LpSAPK9* | MZ345658 | 1143 | 380 | 43.52 | 5.94 | -0.509 |
| 10 | *LpSAPK10* | MZ345653 | 1086 | 361 | 40.68 | 4.79 | -0.284 |

**Supplementary Table 3. The list of protein sequences for phylogenetic tree.**

| Accession no. | Name | Host |
| --- | --- | --- |
| NP_196476.1 | AtSnRK2.1 | *Arabidopsis thaliana* |
| NP_190619.1 | AtSnRK2.2 | *Arabidopsis thaliana* |
| NP_201489.1 | AtSnRK2.3 | *Arabidopsis thaliana* |
| NP_172563.1 | AtSnRK2.4 | *Arabidopsis thaliana* |
| NP_201170.1 | AtSnRK2.5 | *Arabidopsis thaliana* |
| NP_001320129.1 | AtSnRK2.6 | *Arabidopsis thaliana* |
| NP_195711.1 | AtSnRK2.7 | *Arabidopsis thaliana* |
| NP_001077839.1 | AtSnRK2.8 | *Arabidopsis thaliana* |
| NP_179885.1 | AtSnRK2.9 | *Arabidopsis thaliana* |
| NP_849834.1 | AtSnRK2.10 | *Arabidopsis thaliana* |
| ACG50005.1 | ZmSnRK2.1 | *Zea mays* |
| ACG50006.1 | ZmSnRK2.2 | *Zea mays* |
| ACG50007.1 | ZmSnRK2.3 | *Zea mays* |
| ACG50008.1 | ZmSnRK2.4 | *Zea mays* |
| ACG50009.1 | ZmSnRK2.5 | *Zea mays* |
| ACG50010.1 | ZmSnRK2.6 | *Zea mays* |
| ACG50011.1 | ZmSnRK2.7 | *Zea mays* |
| ACG50012.1 | ZmSnRK2.8 | *Zea mays* |
| (Huai et al., 2008) | ZmSnRK2.9 | *Zea mays* |
| ACG50013.1 | ZmSnRK2.10 | *Zea mays* |
| ACG50014.1 | ZmSnRK2.11 | *Zea mays* |
| BAD17997.1 | OsSAPK1 | *Oryza sativa* |
| BAD17998.1 | OsSAPK2 | *Oryza sativa* |
| BAD17999.1 | OsSAPK3 | *Oryza sativa* |
| BAD18000.1 | OsSAPK4 | *Oryza sativa* |
| BAD18001.1 | OsSAPK5 | *Oryza sativa* |
| BAD18002.1 | OsSAPK6 | *Oryza sativa* |
| BAD18003.1 | OsSAPK7 | *Oryza sativa* |
| BAD18004.1 | OsSAPK8 | *Oryza sativa* |
| BAD18005.1 | OsSAPK9 | *Oryza sativa* |
| BAD18006.2 | OsSAPK10 | *Oryza sativa* |
| AJR27157.1 | BdSAPK1 | *Brachypodium distachyon* |
| AJR27158.1 | BdSAPK2 | *Brachypodium distachyon* |
| AJR27159.1 | BdSAPK3 | *Brachypodium distachyon* |
| AJR27160.1 | BdSAPK4 | *Brachypodium distachyon* |
| AJR27161.1 | BdSAPK5 | *Brachypodium distachyon* |
| AJR27162.1 | BdSAPK6 | *Brachypodium distachyon* |
| AJR27163.1 | BdSAPK7 | *Brachypodium distachyon* |
| AJR27164.1 | BdSAPK8 | *Brachypodium distachyon* |
| AJR27165.1 | BdSAPK9 | *Brachypodium distachyon* |
| AJR27166.1 | BdSAPK10 | *Brachypodium distachyon* |

**Supplementary table 4. Subcellular localization predicted by pSORT.**

| Gene Name | Predicated subcellular location (affirmativity) | |
| --- | --- | --- |
| *LpSAPK1* | Cytoplasmic (69.6%) | Nuclear (13.0%) |
| *LpSAPK2* | Nuclear (39.1%) | Cytoplasmic (34.8%) |
| *LpSAPK3* | Cytoplasmic (47.8%) | Nuclear (26.1%) |
| *LpSAPK4* | Nuclear (73.9%) | Cytoplasmic (21.7%) |
| *LpSAPK5* | Cytoplasmic (73.9%) | Nuclear (8.7%) |
| *LpSAPK6* | Cytoplasmic (56.5%) | Nuclear (26.1%) |
| *LpSAPK7* | Cytoplasmic (69.6%) | Nuclear (13.0%) |
| *LpSAPK8* | Cytoplasmic (52.2%) | Nuclear (30.4%) |
| *LpSAPK9* | Nuclear (82.6%) | Cytoplasmic (17.4%) |
| *LpSAPK10* | Cytoplasmic (65.2%) | Nuclear (17.4) |
